# Ad libitum fluid intake leads to no leg swelling in male Ironman triathletes – an observational field study

**DOI:** 10.1186/1550-2783-9-40

**Published:** 2012-09-01

**Authors:** Michael Meyer, Beat Knechtle, Jolanda Bürge, Patrizia Knechtle, Claudia Mrazek, Andrea Wirth, Birte Ellenrieder, Christoph Alexander Rüst, Thomas Rosemann

**Affiliations:** 1 Institute of Primary Care and Health Services Research, University of Zurich, Zurich, Switzerland; 2 Gesundheitszentrum St. Gallen, St. Gallen, Switzerland; 3 Orthopaedic Surgery and Traumatology, Kantonsspital St. Gallen, St. Gallen, Switzerland; 4 Facharzt FMH für Allgemeinmedizin, Gesundheitszentrum St. Gallen, Vadianstrasse 26, St. Gallen, 9001, Switzerland

**Keywords:** Peripheral oedemata, Fluid consumption, Renal function, Triathlon

## Abstract

**Background:**

An association between fluid intake and limb swelling has been described for 100-km ultra-marathoners. We investigated a potential development of peripheral oedemata in Ironman triathletes competing over 3.8 km swimming, 180 km cycling and 42.2 km running.

**Methods:**

In 15 male Ironman triathletes, fluid intake, changes in body mass, fat mass, skeletal muscle mass, limb volumes and skinfold thickness were measured. Changes in renal function, parameters of skeletal muscle damage, hematologic parameters and osmolality in both serum and urine were determined. Skinfold thicknesses at hands and feet were measured using LIPOMETER® and changes of limb volumes were measured using plethysmography.

**Results:**

The athletes consumed a total of 8.6 ± 4.4 L of fluids, equal to 0.79 ± 0.43 L/h. Body mass, skeletal muscle mass and the volume of the lower leg decreased (*p* <0.05), fat mass, skinfold thicknesses and the volume of the arm remained unchanged (*p* >0.05). The decrease in skeletal muscle mass was associated with the decrease in body mass (*p* <0.05). The decrease in the lower leg volume was unrelated to fluid intake (*p* >0.05). Haemoglobin, haematocrit and serum sodium remained unchanged (*p* >0.05). Osmolality in serum and urine increased (*p* <0.05). The change in body mass was related to post-race serum sodium concentration ([Na^+^]) (*r* = −0.52, *p* <0.05) and post-race serum osmolality (*r* = −0.60, *p* <0.05).

**Conclusions:**

In these Ironman triathletes, *ad libitum* fluid intake maintained plasma [Na^+^] and plasma osmolality and led to no peripheral oedemata. The volume of the lower leg decreased and the decrease was unrelated to fluid intake. Future studies may investigate ultra-triathletes competing in a Triple Iron triathlon over 11.4 km swimming, 540 km cycling and 126.6 km running to find an association between fluid intake and the development of peripheral oedemata.

## Background

For more than 30 years, scientists have investigated and described the development of peripheral oedemata in endurance athletes. In 1979, Williams *et al.* studied the effect of seven consecutive days of hill-walking on both water balance and water distribution in five subjects who were allowed to drink water *ad libitum*[1]. They described a retention of plasma sodium (Na^+^) and a reduction in packed cell volume and interpreted these findings as a movement of water from the intracellular to the extracellular space and therefore an expansion of the extracellular volume, leading to visible facial and ankle oedemata. Milledge *et al.* conducted in 1982 a similar study where they investigated five male athletes participating in an endurance exercise of five consecutive days of hill-walking [2]. They also described a retention of both plasma Na^+^ and water and a reduction in packed cell volume. Furthermore, they reported that their athletes developed oedemata at the lower leg and supported therefore the conclusion of Williams *et al.* of a movement of water from the intracellular to the extracellular space, leading to an expansion of the extracellular volume and thus leading to peripheral oedemata [1].

In 1999, Fellmann *et al*. investigated whether a chronic expansion of extracellular water, usually observed during prolonged endurance exercise, was associated with an increase in intracellular water space [3]. In contrast to Williams *et al.*[1] and Milledge *et al.*[2], they observed no decrease in intracellular water space while the extracellular water space increased while investigating nine athletes participating in a seven-day endurance race. Total body water, extracellular water and intracellular water space before, within and after the race were measured. They concluded that a prolonged and repeated endurance exercise induced a chronic hyperhydration at both extracellular and intracellular levels, which was related to exercise intensity. Nevertheless, they confirmed that Na^+^ retention was the major factor in the increase of plasma volume.

In 2010, Knechtle *et al.*[4] investigated the association between fluid intake and the prevalence of exercise-associated hyponatremia (EAH) in 11 female ultra-runners during a 100-km ultra-marathon. These athletes were told to drink *ad libitum*. Serum [Na^+^ and total body water remained unchanged despite a loss in body mass. For male 100-km ultra-marathoners, however, a decrease in body mass with a concomitant loss of both skeletal muscle mass and fat mass as well as with an increase of total body water was reported [5]. It was assumed that the increase in total body water might lead to peripheral oedemata. During a multi-stage ultra-endurance run over 1,200 km, a continuous accumulation of total body water in 10 male ultra-marathoners was shown [6]. It was assumed that the eccentric load of running led to rhabdomyolysis and therefore to an impaired renal function thus leading to a reduced water excretion as the reason for the accumulation of total body water.

In a recent field study, the changes in body mass and fluid metabolism in Triple Iron ultra-triathletes covering 11.4 km swimming, 540 km cycling and 126.6 km running were investigated [7]. Unlike in a marathon, there is a change in sport disciplines in a Triple Iron ultra-triathlon and there is also a high eccentric stress situation due to the 126.6 km of running at the end of the race. The authors reported a decrease in body mass due to both a reduced fat mass and a reduced skeletal muscle mass but not due to dehydration. Furthermore, the development of oedemata after an ultra-endurance performance, such as a Triple Iron ultra-triathlon, has recently been described in a case report [8]. These authors described a persistent increase in the total body water within 42 hours after finishing the race. They concluded, that the remarkably higher fluid intake during the race combined with an impairment of renal function due to muscle damage led to clinically visible oedemata of the feet, persisting for four days post-race. We may assume that comparable to the study from Milledge *et al.*[2] describing oedemata at the lower leg during the prolonged exercise of hill-walking, a Triple Iron ultra-triathlon also leads to oedemata at the lower leg.

There are several different mechanisms, which might lead to a retention of total body water. Maughan *et al.*[9] described an increased plasma volume following an increased protein synthesis. Mischler *et al.*[10] confirmed it in their study measuring the albumin synthetic rates as well as plasma volume and total body water before and after an ultra-endurance trial in six young men. They explained that due to its colloid osmotic properties, albumin mass expansion is the major driving force for plasma volume expansion. On the contrary, Lehmann *et al.*[11] showed that protein catabolism could lead to hypoproteinemic oedemata. A further mechanism was reported by Uberoi *et al.*[12] describing that skeletal muscle damage with severe rhabdomyolysis could lead to an impaired renal function. Furthermore, due to an increased activity of aldosterone the Na^+^ retention increases [3] which therefore results in an increase in plasma volume [2,13].

The quantification of changes in volume of body parts and the development of oedemata is a technical problem. There are different methods described in the literature for quantifying a change in limb volume. Lund-Johansen *et al.*[14] measured the displaced water by weighing whereas Bracher *et al.*[15] used plethysmography, which is quite similar to Lund-Johansen *et al.*[14] method with the difference that using plethysmography the displaced water is quantified as a volume. For a non-invasive examination of the thickness of subcutaneous adipose tissue, the LIPOMETER® has been used in several studies, described as a quick and precise measurement that can be used at any side of the human body [16-18].

Long-distance races such as single ultra-runs [5,19] or multiday runs [6,20] are continuously gaining in popularity all over the world. Especially the 100-km ultra-marathon is one of the most popular distances [21] and therefore, there have already been several studies investigating 100 km runners for changes in body fluid homeostasis [4,22] and development of oedemata [15]. Bracher *et al.*[15] concluded in their study that fluid overload was the most likely aetiology for the increase in limb volumes in 100-km ultra-marathoners. Fluid overload was also frequently reported in Ironman triathlons and as the number of participants in Ironman triathlon is rapidly increasing, studies in this field have become more significant for researchers due to the increasing demand for information [23,24]. Speedy *et al.* showed that fluid overload could also occur in Ironman triathletes, leading to EAH [23]. In the 2000 South African Ironman triathlon, Sharwood *et al.*[25] measured body weight changes, Na^+^ levels and the performance of the participants. The two major findings were that (*i*) the percentage change in body weight was linearly and inversely related to post-race serum [Na^+^ and (*ii*) they reasoned that the low incidence of EAH was due to a conservative drinking policy. No study, however, has investigated a potential development of oedemata in the limbs in Ironman triathletes even though they also bear the risk of fluid overload.

Therefore, we intended to investigate (*i*) whether peripheral oedemata occurred in Ironman triathletes and (*ii*) whether a potential development of peripheral oedemata was due to fluid overload or due to an impaired renal function. The aims of this study were to investigate in male Ironman triathletes (*i*) a potential increase of both the limb volumes and the thickness of the adipose subcutaneous tissue of both hand and feet and (*ii*) in case of an increase in limb volumes and thickness of adipose subcutaneous tissue whether fluid overload or an impairment of renal function was associated with these increases. Fluid overload needs to be distinguished in (*i*) aggressive drinking at a rate greater than water excretion rate, and (*ii*) drinking in response to increased osmolality due to the inflammation products of the prolonged exercise. We hypothesized (*i*) that an Ironman triathlon may lead to an increase of limb volumes or increase the thickness of adipose subcutaneous tissue of the hands and feet as it has been reported for 100-km ultra-marathoners. In case of an increase of limb volumes or thickness of adipose subcutaneous tissue of the hands and feet we hypothesized (*ii*) that the increase was associated with fluid overload.

## Methods

An observational field study at the ‘IRONMAN SWITZERLAND’ in the 2010 race was used for this research.

### Subjects

The organiser of the ‘IRONMAN SWITZER-LAND’ contacted all participants of the 2010 race three months before the start of the race using a separate newsletter and informed them about the planned investigation. A total of 15 recreational male Ironman triathletes volunteered to participate in the study; they all finished the race successfully within the time limit. The characteristics of their anthropometry and training are represented in Table 1. The study was approved by the Institutional Review Board for the Use of Human Subjects of the Canton of Zurich, Switzerland, and all athletes gave their informed written consent.

**Table 1:** **Characteristics of the subjects (*n*** **= 15). Results are presented as mean ± SD**

	**Result**
Age (years)	40.1 ± 6.8
Body mass (kg)	71.3 ± 9.3
Body height (m)	1.75 ± 0.05
Body mass index (kg/m^2^)	23.0 ± 2.2
Years of pre-race experience	7.4 ± 4.9
Weekly swimming kilometres (km)	6.3 ± 2.8
Weekly swimming hours (h)	2.8 ± 1.5
Speed in swimming during training (km/h)	3.2 ± 0.4
Weekly cycling kilometres (km)	202.3 ± 81.5
Weekly cycling hours (h)	7.8 ± 3.0
Speed in cycling training (km/h)	28.5 ± 2.7
Weekly running kilometres (km)	43.5 ± 16.0
Weekly running hours (h)	3.8 ± 1.1
Speed in running during training (km/h)	12.0 ± 1.7

### The race

A total of 2,203 male Ironman triathletes from 49 countries started in the morning at 07:00 a.m. At the start, the air temperature was 14°Celsius and the water temperature in Lake Zurich was 20°Celsius. Wetsuits were allowed due to the low water temperature. At the start, the sky was clear and became cloudy slowly during the afternoon and evening. The highest temperature, 23.2°Celsius, was reached in the afternoon. Humidity was at 69% in the morning and dropped to 37% in the afternoon. Barometric pressure was at 1021.5 hPa at the start and rose to 1014.9 hPa in the afternoon.

The athletes had to swim two laps in the ‘Lake Zurich’ to cover the 3.8 km distance, and then had to cycle two laps of 90 km each, followed by running four laps of 10.5 km each. In the cycling part, the highest point to climb from Zurich (400 metres above sea level) was the ‘Forch’ (700 metres above sea level), while the running course was flat in the City of Zurich. Nutrition was provided for the cycling and running courses by the organisers. They offered bananas, energy bars, energy gels and carbohydrate drinks as well as caffeinated drinks and water on the cycling course. On the running course, in addition to the aforementioned nutrition, different fresh fruits, dried fruits, nuts, chips, salt bars and soup were provided.

### Measurements and calculations

Upon inscription to the investigation, the participants were instructed to keep a training diary until the start of the race. All training units in swimming, cycling and running were recorded, showing distance in kilometres and duration.

The day before the start of the race body mass, body height, the circumferences of the mid-upper arm, mid-thigh, and mid-calf and the thicknesses of eight skin-folds (*i.e.* pectoral, subscapular, axillar, mid-upper arm, abdominal, suprailiacal, mid-thigh, and mid-calf) were measured on the right side of the body in an upright position. With this data, the sum of skin-folds, fat mass and skeletal muscle mass, using an anthropometric method, were estimated.

Body mass was measured using a commercial scale (Beurer BF 15, Beurer GmbH, Ulm, Germany) to the nearest 0.1 kg after voiding of the urinary bladder. Body height was determined using a stadiometer (Tanita HR 001 Portable Height Measure, Tanita Europe, Amsterdam, Netherlands) to the nearest 1.0 cm. The circumferences and the lengths of the limbs were measured using a non-elastic tape measure (cm) (KaWe CE, Kirchner und Welhelm, Germany) to the nearest 0.1 cm. The circumference of the upper arm was measured at mid-upper arm; the circumference of the thigh was taken at mid-thigh and the circumference of the calf was measured at mid-calf. The skin-fold data were obtained using a skin-fold calliper (GPM-Hautfaltenmessgerät, Siber & Hegner, Zurich, Switzerland) and recorded to the nearest 0.2 mm. The skin-fold calliper measures with a pressure of 0.1 Mpa ± 5% over the whole measuring range. The skin-fold measurements were taken following the standard of the International Society for the Advancement of Kinanthropometry (ISAK) once for all four skin-folds and then the procedure was repeated twice more by the same investigator; the mean of the three times was then used for the analyses. The timing of the taking of the skin-fold measurements was standardised to ensure reliability. According to Becque *et al.*[26] readings were performed 4 s after applying the calliper. One trained investigator took all the skin-fold measurements as inter-tester variability is a major source of error in skin-fold measurements. An intra-tester reliability check was conducted on 27 male athletes prior to testing [27]. The intra-class correlation (ICC) within the two measurers was excellent for all anatomical measurement sites, and various summary measurements of skin-fold thicknesses (ICC >0.9). Agreement tended to be higher within measurers than between measurers but still reached excellent reliability (ICC >0.9) for the summary measurements of skin-fold thicknesses.

Fat mass was estimated using the equation following Stewart and Hannan [28] for male athletes: $\text{Fat mass}\left(\text{g}\right)=331.5\times \text{abdominal skin}-\text{fold thickness}+356.2\times \text{thigh}\text{skin}-\text{fold thickness}+111.9\times \text{body mass}–9,108.$Skeletal muscle mass (kg) was estimated using the anthropometric equation of Lee *et al.*[29] with skeletal muscle $\text{mass}=\text{Ht}\times \left(0.00744\times {\text{CAG}}^{2}+0.00088\times {\text{CTG}}^{2}+0.00441\times {\text{CCG}}^{2}\right)+2.4\times \text{sex}–0.048\times \text{age}\;+\;\text{race}\;+\;7.8$ where Ht = height, CAG = skin-fold-corrected upper arm girth, CTG = skin-fold-corrected thigh girth, CCG = skin-fold-corrected calf girth, sex = 1 for male; age is in years; race = 0 for white men and 1 for black men.

The volume and the changes of volume of the right arm and the right lower leg were measured using plethysmography. We used a vessel of plexiglass with the internal dimensions of 386 mm length and 234 mm width. These dimensions were chosen so that any foot size of a male runner would fit in the vessel. The vessel was then filled to 450 mm with plain water and the limb was immersed. For the lower limb, the upper limit of the water was at the middle of the knee; for the upper limb, the upper limit of the water was at the armpit after immersion. The water level was then measured to the nearest 1 mm and the corresponding volume calculated using the length, width and height in millimetres of the displaced water and defined as the volume of the arm and the lower leg, respectively. Cubic millimetres were then converted to litres. The reproducibility of the applied method of water displacement using the changes in height in mm was evaluated in a separate series of 20 consecutive measurements in one individual. The coefficient of variance (CV) was 1.9%; the mean height of displaced water was 12.0 mm, the 95% confidence interval was 11.8-12.1 mm, and the standard error was 0.05. The CV of the pre-race measurements (*n* = 15) was 20.3%, the CV of the post-race measurements was 20.6%.

The thickness of subcutaneous adipose tissue was measured at six sites to the nearest 0.1 mm using LIPO-METER® in an upright position as described by Jürimäe *et al. *[16]. In order to detect an increase in the thickness of the subcutaneous adipose tissue due to a clinically visible or palpable oedemata in the face and limbs [1], the thickness of subcutaneous adipose tissue at the right side of the body at zygomatic arch, the middle of third metacarpal, at the medial border of the tibia, one handbreadth above medial malleolus, directly at medial malleolus and at medial cuneiform was measured*.*

Pre- and post-race, venous blood samples were drawn and urine samples were collected. Two Sarstedt S-Monovettes (plasma gel, 7.5 ml) for chemical and one Sarstedt S-Monovette (EDTA, 2.7 ml) (Sarstedt, Nümbrecht) for haematological analysis were drawn the afternoon before the start of the race and upon arrival at the finish line. Monovettes for plasma were centrifuged at 3,000 g for 10 min at 4 °Celsius. Plasma was collected and stored on ice. Urine was collected in Sarstedt monovettes for urine (10 ml). Blood and urine samples were transported immediately after collection to the laboratory and were analysed within six hours. Immediately after arrival at the finish line, identical measurements were applied. In the venous blood samples, haemoglobin, haematocrit, [Na^+^], [K^+^], creatinine, urea, and osmolality were measured. Hematologic parameters were determined using ADVIA® 120 (Siemens Healthcare Diagnostics, Deerfield, IL, USA). Plasma parameters were measured using COBAS INTEGRA® 800 (Roche, Mannheim, Germany). Osmolality of plasma and urine samples was determined using Fiske® Modell 210 Mikro-Osmometer (IG Instrumenten-Gesellschaft AG, Zurich, Switzerland). In the urine samples, creatinine, urea, [Na^+^], [K^+^], urine specific gravity and osmolality were determined. Specific gravity was analysed using Clinitek Atlas**®** Automated Urine Chemistry Analyzer (Siemens Healthcare Diagnostics, Deerfield, IL, USA). Creatinine and urea were measured using COBAS INTEGRA**®** 800. Electrolytes were determined using ISE IL 943 Flame Photometer (GMI, Inc., Ramsey, MN, USA).

Fractional sodium excretion (FE_Na_) was calculated using the equation ${\text{FE}}_{\text{Na}}=\left(\left({\text{Sodium}}_{\text{Urine}}\times {\text{Creatinine}}_{\text{Plasma}}\right)/\left({\text{Sodium}}_{\text{Plasma}}\times {\text{Creatinine}}_{\text{Urine}}\right)\right)\times 100$ according to Steiner [30]. Fractional urea excretion (FE_Urea_) was calculated using the equation ${\text{FE}}_{\text{Urea}}=\left(\left({\text{Urea}}_{\text{Urine}}\times {\text{Creatinine}}_{\text{Plasma}}\right)/\left({\text{Urea}}_{\text{Plasma}}\times {\text{Creatinine}}_{\text{Urine}}\right)\right)\times 100$ following Dole [31]. Transtubular potassium gradient (TTPG) was calculated using the equation $\text{TTPG}=(\left({\text{Potassium}}_{\text{Urine}}\times {\text{Osmolality}}_{\text{Plasma}}\right)/\left({\text{Potassium}}_{\text{Plasma}}\times {\text{Osmolality}}_{\text{Urine}}\right)$ according to West *et al.*[32]. Creatinine clearance was calculated according Gault *et al.*[33]. Percentage change in plasma volume was determined following Strauss *et al.*[34].

The area of the investigators was located a few meters near the finish line. Immediately after arrival at the finish line the identical measurements were repeated. At the same time, the athletes completed a questionnaire about their intake of solid food and fluids. The investigator prepared a paper where each aid station with the offered food and fluids were indicated. The athletes marked the kind as well as the amount of food and fluid consumed at each aid station. They also recorded additional food and fluid intake provided by the support crew as well as the intake of salt tablets and other supplements. The composition of fluids and solid food were determined according to the reports of the athletes using a food table [35].

### Statistical analysis

Data are presented as mean values ± standard deviation (SD). Pre- and post-race results were compared using paired *t*-test. Pearson correlation analysis was used to check for associations between the measured and calculated parameters. Statistical significance was accepted with *p* <0.05 (two-sided hypothesis).

## Results

The 15 athletes finished the Ironman triathlon within 669.1 ± 79.0 min. They invested 74.4 ± 9.2 min for the swim split, 337.9 ± 33.8 min for the bike split and 247.4 ± 43.0 min for the marathon. Their mean race speed was 3.1 ± 0.4 km/h in swimming, 32.2 ± 3.1 km/h in cycling and 10.5 ± 1.8 km/h in running.

### Fluid and electrolyte intake

While competing, they consumed a total of 8.6 ± 4.4 L of fluids, equal to 0.79 ± 0.43 L/h. Regarding the intake of electrolytes, they consumed 4.1 ± 1.6 g of Na^+^ and 3.7 ± 4.1 g of K^+^, corresponding to 378 ± 151 mg Na^+^ per hour and 330 ± 220 mg K^+^ per hour, respectively.

### Changes in body composition and laboratory results

**Figure 1:**
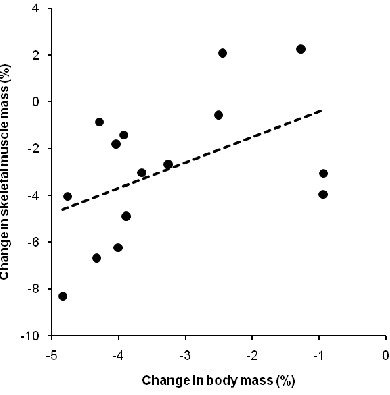
**The change in skeletal muscle mass was significantly and positively related to the change in body mass (*n*** **= 15) (*r*** **= 0.63,*p*** **= 0.012)**.

Table 2 presents the changes in the anthropometric characteristics. Body mass decreased by 2.4 ± 1.1 kg (*p* <0.05). Estimated fat mass, all single skin-fold thicknesses and the sum of eight skin-folds remained unchanged (*p* >0.05). Estimated skeletal muscle mass decreased by 1.2 ± 1.2 kg (*p* <0.05). The volume of the lower leg decreased significantly (*p* <0.05) whereas the volume of the arm remained unchanged (*p* >0.05). The circumferences of thigh and calf decreased (*p* <0.05) whereas the circumference of the upper arm remained unchanged (*p* >0.05). The thickness of the adipose subcutaneous tissue decreased at the medial border of the tibia (*p* <0.05) but remained unchanged at the other sites (*p* >0.05). The decrease of the volume of the lower leg was not associated with the decrease in skeletal muscle mass (*p* >0.05). The change in the lower leg volume was not related to the change in calf circumference (*p* >0.05). The decrease in estimated skeletal muscle mass was associated with the decrease in body mass (*p* <0.05) (Figure 1). Table 3 presents the changes in the laboratory results. Haemoglobin, haematocrit, serum [Na^+^] and serum [K^+^] remained unchanged (*p* >0.05). Plasma volume decreased by 0.4 ± 8.8% (*p* <0.05). Serum creatinine, serum urea and serum osmolality increased (*p* <0.05). Urine specific gravity and urine osmolality increased (*p* <0.05). FE_Na_, FE_Urea_ and creatinine clearance decreased (*p* <0.05). The potassium-to-sodium ratio in urine and TTPG increased (*p* <0.05).

**Table 2:** **Results of the physical parameters before and after the race (*n*** = 15). Results are presented as mean ± SD. * = ***p*<0.05**

	**Pre-race**	**Post-race**	**Absolute change**	**Percent change**
Body mass (kg)	71.3 ± 9.3	68.9 ± 8.8	- 2.4 ± 1.1 *	- 3.2 ± 1.3 *
Circumference of upper arm (cm)	29.8 ± 2.7	29.3 ± 1.8	- 0.5 ± 1.1	- 1.2 ± 3.7
Circumference of thigh (cm)	54.5 ± 4.4	53.0 ± 4.0	- 1.5 ± 2.1 *	- 2.7 ± 3.5 *
Circumference of calf (cm)	37.5 ± 2.2	36.5 ± 1.9	- 1.0 ± 1.3 *	- 2.4 ± 3.6 *
Skin-fold pectoral (mm)	5.8 ± 3.3	5.8 ± 3.1	- 0.0 ± 1.7	- 10.0 ± 45.5
Skin-fold axillar (mm)	8.0 ± 3.3	7.6 ± 3.2	- 0.4 ± 1.0	- 4.8 ± 14.2
Skin-fold triceps (mm)	6.2 ± 2.7	7.0 ± 2.8	+ 0.5 ± 1.6	+ 11.7 ± 29.1
Skin-fold subscapular (mm)	9.3 ± 3.8	9.2 ± 3.2	- 0.1 ± 1.0	- 1.6 ± 10.7
Skin-fold abdominal (mm)	10.2 ± 5.3	11.1 ± 6.0	+ 0.9 ± 1.6	+ 8.5 ± 12.9
Skin-fold suprailiacal (mm)	12.6 ± 7.0	12.3 ± 6.6	- 0.3 ± 3.6	- 1.4 ± 22.9
Skin-fold thigh (mm)	9.4 ± 6.3	9.7 ± 6.6	+ 0.3 ± 1.8	+ 1.6 ± 17.0
Skin-fold calf (mm)	4.6 ± 2.9	4.1 ± 1.8	- 0.5 ± 1.5	- 0.7 ± 23.9
Sum of eight skin-folds (mm)	66.3 ± 30.1	66.8 ± 29.5	+ 0.5 ± 5.0	+ 1.5 ± 8.0
Estimated fat mass (kg)	5.6 ± 4.4	5.7 ± 4.7	+ 0.1 ± 0.9	+ 2.4 ± 15.0
Estimated skeletal muscle mass (kg)	38.9 ± 3.5	37.7 ± 2.6	- 1.2 ± 1.2 *	- 2.9 ± 3.0 *
Volume of the lower leg (L)	3.85 ± 0.50	3.61 ± 0.44	- 0.24 ± 0.25 *	- 5.86 ± 6.86 *
Volume of the arm (L)	2.33 ± 0.44	2.41 ± 0.45	+ 0.08 ± 0.49	+ 6.15 ± 26.06
Thickness subcutaneous fat at zygomatic arch (mm)	3.56 ± 1.97	2.92 ± 1.14	- 0.64 ± 1.18	- 9.1 ± 30.7
Thickness subcutaneous fat at third metacarpal (mm)	2.92 ± 1.54	2.20 ± 0.86	- 0.72 ± 1.99	- 3.5 ± 78.0
Thickness subcutaneous fat at medial border of the tibia (mm)	2.82 ± 0.73	3.39 ± 1.04	+ 0.56 ± 0.82 *	+ 22.1 ± 29.5 *
Thickness subcutaneous fat at medial malleolus (mm)	3.06 ± 1.15	3.58 ± 1.32	+ 0.52 ± 1.49	+ 28.1 ± 54.5
Thickness subcutaneous fat at medial cuneiform (mm)	2.04 ± 1.08	2.29 ± 1.08	+ 0.25 ± 1.57	+ 37.2 ± 92.7

**Table 3:** **Results of the haematological and urinary parameters before and after the race (*n*** = 15). Results are presented as mean ± SD. * = ***p*<0.05**

	**Pre-race**	**Post-race**	**Absolute change**	**Percent change**
Haemoglobin (g/dl)	14.8 ± 0.7	15.0 ± 0.9	+ 0.2 ± 0.6	+ 1.2 ± 4.3
Haematocrit (%)	43.9 ± 2.5	43.7 ± 2.9	- 0.2 ± 2.6	- 0.4 ± 5.8
Serum sodium (mmol/l)	138.9 ± 1.4	140.0 ± 2.9	+ 1.1 ± 2.9	+ 0.8 ± 1.8
Serum potassium (mmol/l)	4.4 ± 0.4	4.4 ± 0.4	+ 0.0 ± 0.5	+ 0.7 ± 12.0
Serum creatinine (μmol/l)	76.3 ± 9.2	94.5 ± 19.1	+ 18.2 ± 19.6 *	+ 25.2 ± 30.0
Serum urea (mmol/l)	5.9 ± 1.1	9.0 ± 1.1	+ 3.1 ± 1.2 *	+ 57.6 ± 27.6
Serum osmolality (mosmol/kgH_2_O)	296.6 ± 2.9	304.6 ± 6.0	+ 8.0 ± 6.3 *	+ 2.7 ± 2.1
Urine specific gravity (g/ml)	1.013 ± 0.006	1.026 ± 0.005	+ 0.013 ± 0.007 *	+ 1.33 ± 0.76
Urine osmolality (mosmol/kgH_2_O)	531.7 ± 271.2	836.5 ± 196.3	+ 304.8 ± 201.3 *	+ 94.5 ± 88.9
Fractional sodium excretion (%)	1.32 ± 0.76	0.39 ± 0.27	- 0.93 ± 0.65 *	- 66.6 ± 23.1
Fractional urea excretion (%)	54.2 ± 10.9	29.2 ± 11.7	- 25.0 ± 14.2 *	- 44.6 ± 23.1
Creatinine clearance (ml/min)	116.5 ± 23.4	91.6 ± 15.5	- 24.9 ± 25.7 *	- 19.3 ± 16.0
Potassium-to-sodium ratio in urine (ratio)	0.54 ± 0.40	4.41 ± 4.96	+ 3.87 ± 4.88 *	+ 996 ± 1,504
Transtubular potassium gradient (ratio)	22.4 ± 17.8	100.1 ± 60.3	+ 77.7 ± 59.2 *	+ 936 ± 1,230

### Correlations between fluid intake and changes in body composition

**Figure 2:**
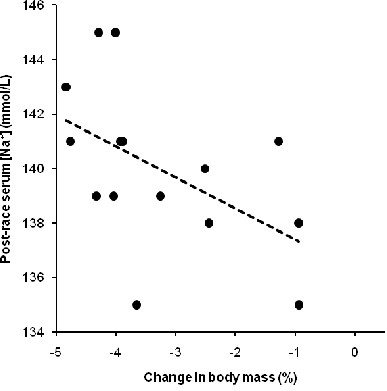
**The change in body mass was significantly and negatively related to post-race serum [Na**^**+**^**] (*n*** **= 15) (*r*** **= −0.52,*p*** **= 0.045)**.

**Figure 3:**
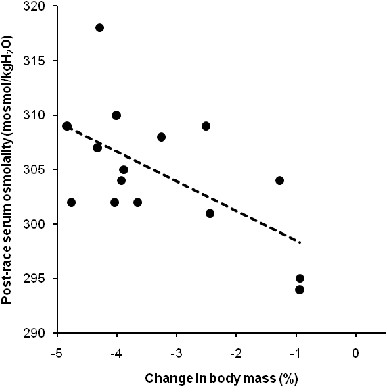
**The change in body mass was significantly and negatively related to post-race serum osmolality (*n*** **= 15) (*r*** **= −0.60,*p*** **= 0.017)**.

Fluid intake was unrelated to the decrease in body mass (*p* >0.05). The change in body mass was not associated with the change in serum [Na^+^] (*p* >0.05). The change in body mass was related to both post-race serum [Na^+^] (Figure 2) and post-race serum osmolality (Figure 3) (*p* <0.05). The decrease of the volume of the lower leg was unrelated to fluid intake (*p* >0.05). Fluid intake was neither related to the changes in the thickness of adipose subcutaneous tissue nor to the changes in skin-fold thicknesses (*p* >0.05). Sodium intake was not related to post-race serum [Na^+^] (*p* >0.05). Post-race serum [Na^+^] was unrelated to both the change in the potassium-to-sodium ratio in urine and TTKG (*p* >0.05). The increase in serum urea was not related to the increase in serum osmolality (*p* >0.05). The change in serum urea was unrelated to the change in skeletal muscle mass (*p* >0.05). The change in the thickness of the adipose subcutaneous tissue at the medial border of the tibia was significantly and positively associated with the change in creatinine clearance (*r* = 0.58, *p* = 0.025). The increase in the thickness of adipose subcutaneous tissue at the medial border of the tibia was not related to the non-significant change in skin-fold thickness of the calf (*p* >0.05). The non-significant changes in skin-fold thicknesses were neither related to overall race time nor to the split times (*p* >0.05).

## Discussion

In the present study, we investigated potential changes in both limb volumes and thicknesses of adipose subcutaneous tissue at the extremities in male Ironman triathletes in order to quantify a potential development of peripheral oedemata. We hypothesized that an Ironman triathlon would lead to an increase of both limb volumes and the thicknesses of adipose subcutaneous tissue of the hands and feet as has been shown for 100-km ultra-marathoners. However, we found a significant decrease in the lower leg volume, unrelated to both the decrease in body mass and skeletal muscle mass. Haemoglobin, haematocrit and serum [Na^+^] remained unchanged indicating that no fluid overload occurred. The sum of eight skin-folds remained unchanged showing that no increase in the thickness of the subcutaneous adipose tissue occurred. Plasma [Na^+^] and plasma osmolality were maintained showing that body fluid homeostasis remained unchanged.

### Decrease in lower leg volume but not in arm volume

The most important finding regarding the question of developing peripheral oedemata in Ironman triathletes was that the volume of the lower leg decreased and the decrease in the lower leg volume was unrelated to fluid intake. Regarding the findings from Milledge *et al.*[2], Knechtle *et al.*[8] and Bracher *et al.*[15] all describing a development of oedemata after a prolonged endurance performance, we expected to find also after an Ironman triathlon an increase in the lower leg volume, but not a decrease. However, these Ironman triathletes showed no swelling of the lower leg where a possible explanation for the decrease in the lower limb volume could be a loss in skeletal muscle mass [36]. However, since the change in skeletal muscle mass showed no association with the decrease in lower leg volume, this explanation is unlikely. In contrast to the present findings, Bracher *et al.*[15] also found a relationship between fluid intake and changes in both arm and lower leg volumes in 100-km ultra-marathoners. Since they reported no association between endocrine and renal parameters with the changes in limb volumes, they concluded that fluid overload was the most likely mechanism leading to an increase in the limb volumes. In the present Ironman triathletes, no fluid overload occurred, which therefore could be an explanation why the volume of the lower leg showed no increase and why we found no relationship between fluid intake and the change in the lower leg volume.

### Maintenance of body fluid homeostasis

A further important finding was that serum [Na^+^ remained unchanged and serum osmolality increased whereas total body mass significantly decreased. These findings support the recent results of Tam *et al.*[37] reporting that the body primarily defends both plasma [Na^+^ and plasma osmolality and not body mass during both a 21.1-km and a 56-km foot race. Furthermore, fluid intake showed no association with the change in body mass. Since haemoglobin, haematocrit and serum [Na^+^ were kept constant, body fluid homeostasis remained unchanged. This is most likely because these Ironman triathletes did not overdrink and no fluid overload occurred. Noakes *et al.*[38] described that fluid overload as a consequence of excessive drinking, correlated with both a decrease in serum [Na^+^ and an increase in body mass. This has also been confirmed by Noakes *et al.*[39] and Speedy *et al.*[40] where Ironman athletes with less weight loss showed a lower serum [Na^+^. This leads us to the conclusion that in the present Ironman triathletes no fluid overload occurred and therefore no disturbance of the body fluid homeostasis or of any other dimension could be determined.

Fluid overload, as a consequence of excessive drinking, is the main risk factor in the pathogenesis of exercise-associated hyponatremia (EAH) [38,41,42]. Regarding the ‘Position Statement’ of the ‘International Marathon Medical Directors Association’ [43] which recommends drinking *ad libitium* between 0.4 and 0.8 L/h during a race the present Ironman triathletes behaved correctly by drinking only in response to their thirst. Like in the reports of Hew-Butler *et al.*[44], Speedy *et al.*[45], and Noakes [46] describing no correlation between sodium intake, post-race serum [Na^+^ and the change in serum [Na^+^, we also found no correlation between these parameters and therefore can confirm their findings. Kavouras [47] and Shireffs [48] described that in case of dehydration body mass decreases while urine specific gravity increases. In the present Ironman athletes, body mass significantly decreased by 3.2% and urine specific gravity significantly increased by 1.33% indicating dehydration following their definition [47,48].

### Decrease in the circumferences of the lower limb but not of the upper limb

A further finding was that the circumferences of the thigh and the calf decreased by 2.7% and 2.4%, respectively, whereas the circumference of the upper arm remained unchanged. This indicates that the estimated skeletal muscle mass at the lower limbs became reduced. Since the change in the estimated skeletal muscle mass showed no association with the change in plasma urea, we presume that no substantial degradation of myofibrillar proteins must have occurred, and the loss in estimated skeletal muscle mass might be due to a depletion of intramyocellular stored energy, such as muscle glycogen and intramyocellular lipids [49]. We furthermore found a relationship between the change in estimated skeletal muscle mass and the change in body mass. This finding confirms recent findings where Ironman triathletes lost skeletal muscle mass [36]. However, it was unexpected that the decrease in estimated skeletal muscle mass showed no association with the decrease in the lower leg volume.

However, the reduction in limb circumference could also be due to a reduction in interstitial fluid. The decrease in the lower leg volume might also suggest an action of the ‘muscle pump’ during exercise helping to clear pre-race swelling. Perhaps the tapered athlete started with oedemata, the result of being relatively inactive in a pre-race taper. If so, perhaps the muscle pump cleared this oedemata during the race, and perhaps clearing was aided by compression socks. Regarding the results concerning the decrease in the circumferences of both the thigh and the calf, we expected that the main areas of decrease would occur in the muscles used most, meaning in the lower leg and thigh muscles. Because the thigh has a larger skeletal muscle mass than the calf, it is likely that the change in the thigh muscle mass influenced the change in estimated skeletal muscle mass more than the change in calf muscle mass did. Another possible explanation could be that there actually would have been a correlation between the decrease of the lower leg volume and the estimated skeletal muscle mass, but that this correlation was influenced due to a non-quantified change in tissue fluid in the lower leg. As we were using plethysmography for measuring the volumes of the whole limbs, we were not able to differentiate a change in volume between arm and hand or between lower leg and foot, respectively. This could have influenced our results. Lund-Johansen *et al.*[14] measured the displaced water by weighing, which is a similar method to the plethysmography. These authors concluded that water displacement volumetry was a sensitive method for the measurement of leg volume. We therefore think that using plethysmography for measuring the leg volume is a sensitive method as well. Unfortunately, both methods have the limitation of not being able to differentiate between volume changes in the measured compartment or to differentiate between the volume changes of the body composition. For example, if the volume of the lower leg decreases due to a depletion of intramyocellular stored energy while the same amount of volume increases due to oedemata occurring in the skeletal muscle mass or in the adipose subcutaneous tissue, we could not measure any volume change using plethysmography. In previous studies, it was shown that oedemata did not develop immediately with the exercise or the race but shortly afterwards. Knechtle *et al.*[8] measured the highest total body water one day after a Triple Iron ultra-triathlon, Williams *et al.*[1] described a peak water retention on day 5 of consecutive hill-walking and Milledge *et al.*[2] measured the largest gain in the leg volumes one day after five consecutive days of hill-walking. There is inactive time between exercise bouts, no muscle pump, and therefore the possibility for swelling to build. Nor is there any mechanism to decrease swelling on subsequent days.

### Potential correlation between oedemata and renal function?

Another interesting finding was that the change in the thicknesses of adipose subcutaneous tissue at medial border of the tibia was significantly and positively associated with the change in creatinine clearance. However, correlation analysis does not prove cause and effect; therefore this correlation must be questioned. In case of an impairment of renal function, we would expect a development of peripheral oedemata [50,51]. However, the level of renal impairment was trivial in these athletes and would not have produced peripheral oedemata. Nevertheless, we cannot postulate an association between a decrease of the renal function and an increase of the thickness of the adipose subcutaneous tissue of the lower leg. This supports the findings of Bracher *et al.*[15] describing no association between a change in renal parameters and a change in limb volume in 100-km ultra-marathoners and thus concluded that not the change in renal function but rather the fluid overload was the more likely mechanism leading to an increase in limb volumes. Eisenbeiss *et al.*[52] showed, by measuring both the thickness of the dermis and the echodensity using a high-frequency ultrasound, that slight changes in the water distribution of the body could influencing the thickness of the dermis under various physiological conditions.

In the present study, a reason why the thickness of the adipose subcutaneous tissue of the lower leg showed no increase might be due to the compression, which might be induced by wearing socks and running shoes. Knechtle *et al.*[5] also described this phenomenon, where several runners only developed oedemata of the feet after taking of their shoes, decreasing the compression and allowing the fluid to redistribute from the lower leg into the foot, especially into the subcutaneous adipose tissue. Compared to Bracher *et al.*[15] describing an increase in the thickness of adipose subcutaneous tissue at medial malleolus and at medial cuneiform but not at medial border of the tibia or zygomatic arch in 100-km ultra-marathoners, and thus made the conclusion of a redistribution of fluid into the subcutaneous adipose tissue of the hands and feet, we found an increase of the subcutaneous adipose tissue at the medial border of the tibia but no change at any other site. Therefore, we were unable to confirm this hypothesis. The fact that we found only one association between the thickness of the adipose subcutaneous tissue and the creatinine clearance but neither with the other skin-fold thicknesses nor with Fe_Na_ or Fe_Urea_ is also an argument against any association between a change of the adipose subcutaneous tissue and a change in renal function. Fe_Na_ and Fe_Urea_ are parameters which can be used to detect an impairment of the renal function [53,54]. Since correlations are often used in studies it is important to understand the exact meaning and limits of a correlation. A correlation describes a relationship between two or more statistical variables. However, it does not give us any information whether there is a causal relationship between these variables or not.

The present Ironman triathlon with a mean average race time of about eleven hours was rather short when compared to the studies from Milledge *et al.*[2], Williams *et al.*[1] and the case study form Knechtle *et al.*[8] where the races took place over several days. If we also consider the studies from Dancaster *et al.*[50], Irving *et al.*[51] and Knechtle *et al.*[6] showing that a longer eccentric load of running leads to an increased skeletal muscle damage due to rhabdomyolysis, which therefore impairs the renal function and thus leads to a higher water retention [6], the eccentric stress situation in the present Ironman triathletes was comparably low. In addition, the extent of renal impairment in the present Ironman triathletes was minimal which would not have led to peripheral oedemata. Skenderi *et al.*[19] also demonstrated rhabdomyolysis during a 246-km continuous running race and postulated an association between muscle damage and impaired renal function. It has furthermore been described by Uberoi *et al.*[12] that the pathophysiology of acute renal failure is multifactorial and is the combined effect of rhabdomyolysis, dehydration, hypotension, intake of non-steroidal anti-inflammatory drugs and hyperuricemia. Concluding that a longer race time leads to a larger decrease of the renal function due to an increased rhabdomyolysis, we have to assume that the race time of the Ironman triathlon was probably too short to measure a significant disturbance in body fluid homeostasis.

### Venous and lymphatic reasons for post-race oedemata?

The type of oedemata that develops following an Ironman triathlon is not necessarily the result of frank rhabdomyolysis. Leg swelling is often of oedematous nature [55] where bilateral leg swelling is usually the manifestation of a systemic disorder, the most common of which is chronic venous insufficiency [56]. Systemic causes of leg oedema may also include idiopathic cyclic oedema, heart failure, cirrhosis, nephrosis and other hypoproteinemic states [57]. The legs are preferentially affected by systemic oedematous states. Pathogenetic factors are: increased hydrostatic pressure, increased capillary permeability (leak), reduced colloid-oncotic pressure, reduced lymph drainage and miscellaneous rare conditions [58]. The post-race oedemata in these athletes can easily be understood as an interstitial oedema, partly explained by increased capillary permeability, allowing leakage of osmotic material. Peripheral oedemata develop as a consequence of imbalance in the processes of filtration, resorption and lymphatic transport in the capillary bed [59]. Water follows into the interstitium to restore/maintain the osmotic equilibrium. This swelling is cleared by the slow acting lymphatic circulation. The kidneys see this fluid only once the lymphatic circulation returns it to blood vessels. The post-race oedemata of the lower legs in these Ironman triathletes might also be due to these reasons.

It should also be noted that this kind of oedema cannot be said to be due to aggressive overdrinking completely unrelated to thirst. Excess water is ingested because the debris of prolonged exercise increases the osmolality of body water, appropriately increasing thirst. The mean fluid intake in these Ironman triathletes was 0.79 ± 0.43 L/h. In a recent study on 100-km ultra-marathoners showing an association between fluid intake and limb swelling, the athletes consumed 0.63 ± 0.20 L/h [60]. Obviously, the 100-km ultra-marathoners consumed less fluid and developed an association between fluid intake and limb swelling in contrast to the present Ironman triathletes drinking more fluids without a relationship between fluid consumption and lower leg swelling. The pathogenesis of lower limb swelling in ultra-endurance athletes may involve the nature of exercise debris, the increased permeability of the capillaries allowing leakage of osmotic material, the ingestion of water to restore/maintain osmotic equilibrium, and the role of lymphatic circulation in clearing the oedemata. We assume that we cannot reduce the swelling in lower legs in ultra-endurance athletes due to excessive fluid intake.

### Strengths and limitations of the present study and implications for future research

A strength of this study was that anthropometric measurements were performed immediately upon arrival at the finish line. A limitation of the present study was that by measuring the entire lower leg volume, or arm volume, we could not precisely quantify nor locate specifically where the changes in volume occurred*.* An implication for future research would therefore be to measure the volume of hands and feet separately from the arms and the legs using plethysmography. It would as well be useful to have a measurement method that allows us to differentiate the volume changes occurring in a body part into the different body compositions. Bioelectrical impedance analysis [61] for example is a commonly used method for estimating body compositions, although it measures the composition of the whole body and not just of one body part [62]. However, this methodology may not provide valid estimates of total body water when hydration status is altered [63] since plasma osmolality and sodium concentration should be unchanged [64,65]. Regarding the studies from Knechtle *et al.*[9], Milledge *et al.*[2] and Williams *et al.*[1] describing an increase in the mean leg volume not immediately after the endurance performance but shortly afterwards, it would also be appropriate to take another measurement later on after the race. Concluding that race time in these Ironman triathletes was relatively short to disturb the body fluid homeostasis [1,2,6,66] it would furthermore be reasonable for future studies to perform these measurements during a longer race such as a Triple Iron ultra-triathlon [7]. Furthermore, we were not able to determine the effect that non-steroidal anti-inflammatory drugs (NSAIDs) had on the decrease of the renal function because we did not trace the consumption of NSAIDs. These drugs are known to increase the potential effects of vasopressin by inhibiting renal prostaglandin synthesis via the Cyclooxygenase-2 (COX-2) isoform of cyclo-oxygenase [67,68]. NSAIDs decrease the glomerular filtration rate when given to those with effective volume depletion, such as exercising endurance athletes [69]. Hew *et al.*[42] reported that up to 50-60% of the athletes are consuming NSAIDs. Thermal stress in these athletes was mild to moderate; a higher thermal stress might have altered fluid status to a larger extent. A further limitation was that we did not differ between athletes wearing compression socks and athletes without compression socks. A recent study showed that compression socks improved running performance [70] and athletes may nowadays use more frequently compressions socks during races. The use of compression socks might have influenced the post-race volume of the lower leg. Since oedemata develop over the course of multi-day events, it would be interesting to repeat this study for a standard Ironman triathlon conducted in hot weather. It would also be interesting to follow the time course of developing and receding oedemata in multi-stage ultra-marathons. A recent study showed that body mass decreased after each stage and reached pre-race value by the morning of the next day in a multi-stage mountain ultra-marathon [71]. Finally, it would be interesting to chart the time-course of oedemata ‘growing in’ as well as receding in future studies.

## Conclusions

To summarize, the volume of the lower extremity decreased and this decrease was unrelated to fluid intake in the present male Ironman triathletes. We found no increase in the thickness of adipose subcutaneous tissue of the hands and feet. Renal function was altered. Serum [Na^+^ was maintained and serum osmolality increased because body mass decreased. Considering the findings of Milledge *et al.*[2] and Williams *et al.*[1], the duration of an Ironman triathlon was presumably too short to find significant disturbances in body fluid homeostasis. Also the athletes in the race faced only a mild to moderate thermal stress. Future studies on longer triathlon distances such as a Triple Iron ultra-triathlon and races under higher thermal stress may be more appropriate to find a disturbance in body fluid homeostasis leading to peripheral oedemata in triathletes. In these athletes, the prevalence of EAH is considerably higher compared to Ironman triathletes and therefore the risk for fluid overload might be higher [72]. For future studies, peripheral quantitative computed tomography (pqCT) might be used to estimate changes in muscle and fat in the lower leg [73].

## Competing interests

The authors declare that they have no competing interests.

## Authors’ contributions

MM drafted and wrote the manuscript. BK designed the study and assisted the manuscript preparation. BK, JB, PK, CM, AM and BE conducted all the measurements during two field study for data collection before and after the race. CAR and TR assisted in data analyses, statistical analyses, data interpretation and manuscript preparation. All authors have read and approved the final version of the manuscript.
